# Developing Information Technologies to Promote Dementia e-Friendly Communities for COVID-19 and Beyond

**DOI:** 10.1093/geroni/igab046.2428

**Published:** 2021-12-17

**Authors:** Nicole Ruggiano, Yan Luo, Amy Hurd, Kristen Lawlor, Monica Anderson, Zhe Jiang, Jeff Gray

**Affiliations:** 1 University of Alabama, Hoover, Alabama, United States; 2 The University of Alabama, Tuscaloosa, Alabama, United States; 3 University of Alabama, Tuscaloosa, Alabama, United States

## Abstract

People living with dementia (PLWD) and their caregivers often face barriers to education, support, and services that can improve their health and quality of life. Information technology (IT) has been suggested as a solution to overcoming such barriers, though the development of evidence-based IT for dementia care is still developing. This project gathered stakeholder (e.g., providers, caregivers) perspectives on the development of a proposed IT solution to support community asset mapping that would allow families to self-assess their dementia-related service needs, educate them about available services, and link them with services they need in their community. This proposed IT would create a dementia resource database that relies on crowdsourced data from community stakeholders as well as relevant data mined from existing sources (e.g., CMS certified nursing home data). As part of the planning process, this project conducted qualitative interviews with providers and caregivers in four metro areas in Alabama and their surrounding rural communities to learn more about the content and features that stakeholders perceive as being most effective for the proposed technology. Stakeholders also discussed their experience of utilizing IT solutions during the COVID-19 pandemic to promote access and continuum of care when barriers to service intensified. Thematic findings provide detail on: 1) motivating factors among stakeholders to contribute crowdsourced data that support community members affected by dementia; 2) potential barriers to implementing IT for dementia support, based on experiences with IT use during COVID-19; and 3) how stakeholders envision IT to better connect community members with needed services.

